# Safety and efficacy of low-dose PI3K inhibitor taselisib in adult patients with CLOVES and Klippel–Trenaunay syndrome (KTS): the TOTEM trial, a phase 1/2 multicenter, open-label, single-arm study

**DOI:** 10.1038/s41436-021-01290-y

**Published:** 2021-08-12

**Authors:** M. Luu, P. Vabres, H. Devilliers, R. Loffroy, A. Phan, L. Martin, F. Morice-Picard, F. Petit, M. Willems, D. Bessis, M. L. Jacquemont, A. Maruani, C. Chiaverini, T. Mirault, J. Clayton-Smith, M. Carpentier, C. Fleck, A. Maurer, M. Yousfi, V. E. R. Parker, R. K. Semple, M. Bardou, L. Faivre

**Affiliations:** 1 Centre d’Investigation Clinique–module plurithématique, CHU, Dijon, France; 2 INSERM CIC1432, UBFC, Dijon, France; 3 Centre référence MAGEC, Dijon, France; 4 Centre de Référence Anomalies du Développement et Syndromes Malformatifs et FHU TRANSLAD, CHU, Dijon, France; 5 Centre d’Investigation Clinique–module épidémiologie clinique, CHU, Dijon, France; 6 Radiologie Interventionnelle, CHU, Dijon, France; 7 Dermatologie Pédiatrique, HFME, Lyon, France; 8 Centre référence MAGEC, CHU, Angers, France; 9 Centre de référence MRP–Sud, CHU, Bordeaux, France; 10 Centre de référence Anomalies du Développement et Syndromes Malformatifs, CHU, Lille, France; 11 Centre de référence Anomalies du Développement et Syndromes Malformatifs, Montpellier, France; 12 Service de Dermatologie, CHU, Montpellier, France; 13 Centre de Référence Anomalies du Développement et Syndromes Malformatifs, CHU La Réunion, Saint-Pierre, France; 14 Centre référence MAGEC, CHU, Tours, France; 15 Centre de référence MRP–Sud, CHU, Nice, France; 16 Centre de référence maladies vasculaires rares, Hôpital européen Georges-Pompidou, AP-HP, Paris, France; 17 INSERM U970 PARCC, Université de Paris, Paris, France; 18 Clinical Genetics and Manchester Centre for Genomic Medicine, NHS and Manchester University, Manchester, UK; 19 Délégation à la Recherche Clinique et de l’Innovation, CHU, Dijon, France; 20 AstraZeneca, Cambridge, UK; 21 Centre for Cardiovascular Science, University of Edinburgh, Edinburgh, UK; 22 INSERM UMR1231 GAD, Génétique des Anomalies du Développement, Université Bourgogne Franche-Comté, Dijon, France

## Abstract

**Purpose:**

*PIK3CA* pathogenic variants in the PIK3CA-related overgrowth spectrum (PROS) activate phosphoinositide 3-kinase signaling, providing a rationale for targeted therapy, but no drug has proven efficacy and safety in this population. Our aim was to establish the six-month tolerability and efficacy of low-dose taselisib, a selective class I PI3K inhibitor, in PROS patients.

**Methods:**

Patients over 16 years with PROS and *PIK3CA* pathogenic variants were included in a phase IB/IIA multicenter, open-label single-arm trial (six patients at 1 mg/day of taselisib, then 24 at 2 mg/day). The primary outcome was the occurrence of dose limiting toxicity (DLT). Efficacy outcomes were the relative changes after treatment of (1) tissue volume at affected and unaffected sites, both clinically and on imaging; (2) cutaneous vascular outcomes when relevant; (3) biologic parameters; (4) quality of life; and (5) patient-reported outcomes.

**Results:**

Among 19 enrolled patients, 2 experienced a DLT (enteritis and pachymeningitis) leading to early trial termination (17 treated, 10 completed the study). No serious adverse reaction occurred in the 1 mg cohort (*n* = 6). No significant reduction in affected tissue volume was observed (mean −4.2%; *p* = 0.81; SD 14.01). Thirteen (76.4%) participants reported clinical improvement (pain reduction, chronic bleeding resolution, functional improvement).

**Conclusion:**

Despite functional improvement, the safety profile of low-dose taselisib precludes its long-term use.

## INTRODUCTION

PI3KCA-related overgrowth spectrum (PROS) is a group of rare diseases induced by postzygotic activating variants in the *PIK3CA* gene, encoding of the phosphoinositide-4,5-bisphosphate 3-kinase (PI3K) catalytic subunit alpha. The pathogenic variants produce congenital mosaic tissue overgrowth. PROS encompasses several developmental phenotypes, such as congenital lipomatous overgrowth with vascular malformations, epidermal nevi, and scoliosis (CLOVES) syndrome, megalencephaly–capillary malformation (MCAP) syndrome, congenital lipomatous overgrowth, and a large proportion of cases of Klippel–Trenaunay syndrome (KTS) [1, 2]. The PI3K/AKT/mammalian target of rapamycin (mTOR) signaling pathway has been a major cancer target, with several candidate inhibitors investigated in oncology trials. Gain-of-function *PIK3CA* variants in overgrowth syndromes thus provide a strong rationale for targeted PI3K/AKT/mTOR inhibition as a therapeutic strategy in PROS. Unlike in cancer, however, life-threatening complications are uncommon in PROS, which is generally a chronic disease, with the PROS subphenotype largely dependent on the type and location of affected tissue. The dominant concern in PROS is mass effects of overgrowth, which may result in functional impairment, or compression of surrounding unaffected tissue. Serial, invasive debulking surgery has been the mainstay of therapy to date.

The repertoire of postzygotic *PIK3CA* pathogenic variants in PROS is identical to that in cancer, and so repurposing of candidate anticancer drugs targeted at PI3K is an obvious strategy. The path of such repurposed pharmacological treatments in PROS is promising to date, but raises the urgent need for assessment of long-term safety and efficacy. This will have to address specific and substantial methodological challenges, including the small size of target populations, the difficulty of defining measurable outcomes in a widely varying syndrome spectrum, and the need to assess dose ranges that limit side effects from long-term use. Most published data come from individual reports on compassionate use of sirolimus, where safety issues have not been fully explored [3–5]. As an example, sirolimus was deemed safe and effective in PROS patients until the PROMISE trial, the first clinical trial in this population. Partial reduction of overgrowth and pain was achieved with sirolimus, but only at the cost of substantial adverse effects [6].

A significant reduction in lymphatic malformation volume and soft tissue hypertrophy has recently been reported in patients treated with the selective PI3K alpha subunit inhibitor alpelisib at standard doses used in cancer, under compassionate use. This was undertaken outside the setting of a registered clinical trial, however, precluding standardized assessment of benefit and risk [7].

Taselisib is a selective class I PI3K inhibitor developed for breast cancer therapy, in which a daily dose of 6 mg has been used [8]. This has been demonstrated to suppress aberrant PI3K hyperactivation in nonclinical pharmacological studies [9]. Our aim was now to investigate the safety and efficacy of low-dose taselisib in PROS adult patients within a phase IB/IIA trial.

## MATERIALS AND METHODS

### Study design and participants

We report on a national multicenter open-label, single-arm, dose escalation phase IB/IIA trial, to evaluate the six-month tolerability of taselisib therapy in PROS patients aged 16 to 65 years old. It was conducted in nine university hospitals in France. Patients were eligible if they had a postzygotic *PIK3CA* variant and functional or cosmetic impairment. Samples used for diagnosis in standard care were collected from biopsies of affected sites, when accessible, or skin biopsies when biopsies of affected sites were not feasible. Exclusion criteria were life-threatening manifestations of PROS in the opinion of the investigator, previous treatment with one or more mTOR/PI3K inhibitor within the last 12 weeks, concomitant treatment with strong CYP3A4 inhibitors, or diseases predisposing to known PI3K inhibitor-related adverse events, such as presence or history of colitis, diabetes and impaired glucose tolerance, or pregnancy. Patients with MCAP were also excluded from the study in the absence of measurable cerebral endpoints. Full inclusion and exclusion criteria are detailed in Table S1.

**Fig. 1:**
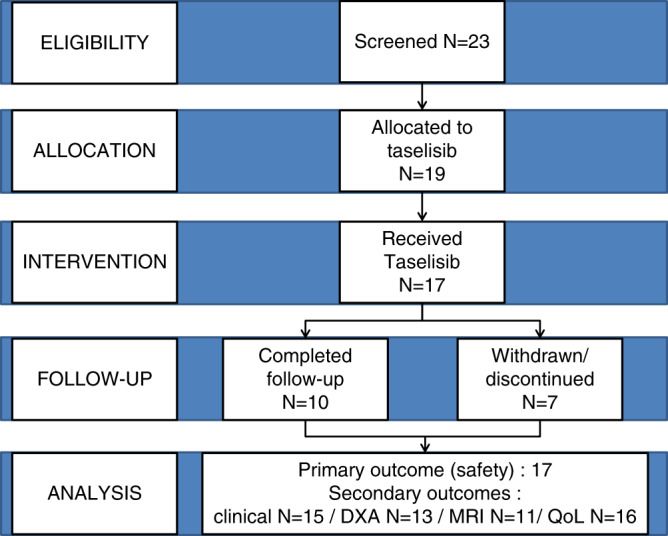
CONSORT flowchart of TOTEM study. On the 19 subjects enrolled, 10 completed 26 weeks of taselisib therapy. All treated patients were analyzed for the primary outcome measure (safety), and 13/19 had anatomy that permitted analysis of the efficacy measure with DXA. DXA dual energy X-ray absorptiometry, MRI magnetic resonance imaging, QoL quality of life.

Two cohorts were scheduled to receive 1 or 2 mg daily of taselisib. Treatment was started at 1 mg/day and continued at the same dose in the first cohort or escalated to 2 mg/day in the second cohort, using a 3 + 3 design with its “rolling 6” extension (see Fig. 1) [10, 11]. In a classical 3 + 3 design, accrual is suspended between batches of three patients, allowing for assessment of dose limiting toxicity (DLT) before enrolling the next dose level. With this extension design, three more patients are needed after the first batch of three patients before allowing DLT assessment, and dose escalation if relevant. It was then intended to include six evaluable participants in the 1 mg/day cohort and at least six others in the 2 mg/day cohort, to permit tolerability to be established. The trial was to be interrupted in case of occurrence of (1) one suspected unexpected serious adverse reaction (SUSAR) of sufficient severity (life-threatening event, invalidity/incapacity, or congenital anomaly); (2) ≥2 SUSAR; (3) 5 serious adverse events [SAE] related to taselisib; (4) 5 or more adverse events (AE) of grade III related to taselisib, after adjudication by an independent data safety monitoring board (DSMB) composed of three independent experts.

The study was performed in accordance with the Declaration of Helsinki and Good Clinical Practice Guidelines (ICH E6). Study was approved by the French ethics review board (Comité de Protection des Personnes [CPP] Ouest V Rennes [ref: 17/017-1]) and the Agence de Sécurité du Médicament et des Produits de Santé (ANSM). Written informed consent and parental consent for minor participants were obtained and archived from all participants. The same applies for clinical photographs taken before and after treatment.

### Outcomes

The primary endpoint was occurrence of DLT, defined as a drug-related toxicity of at least grade 3 occurring in the first month of treatment, using the National Cancer Institute (NCI) Common Terminology Criteria for Adverse Events (CTCAE–Version 4.0). Secondary endpoints included preliminary efficacy, determination of pharmacokinetic parameters, quality of life, biologic parameters.

### Quantification of overgrowth

Affected sites were first measured by a measuring tape at baseline and at evaluation visit by the same physician. Each affected site was measured three times, and the mean value of the three measures was conserved. Efficacy was quantified as the percentage change in volume of measured affected and unaffected areas after treatment compared to baseline. Fat, lean, and total (fat plus lean) tissue volumes were determined using both dual energy X-ray absorptiometry (DXA) and T1-weighted magnetic resonance imaging (MRI) scanning, where anatomically feasible. We used the same methodology as in our previously published PROMISE trial [6]. Affected sites were defined by the clinical observation of either overgrowth, or the presence of skin or vascular abnormalities. Between one and three affected sites per patient (seven patients had one site, three patients had two sites, and one patient had three sites measured) had their volumes measured by MRI. Unaffected sites compared with affected sites were (in order of preference) the contralateral limb/truncal region, a limb or trunk on the same side, or any other site without clear involvement. Those with multifocal, but asymmetric overgrowth were deemed to have no unaffected site.

DXA scans were all performed at the coordinating center, using the same orientation for each participant at 0 and 26-week time points. Soft tissue volumes were obtained for total body and various body segments (left leg, right leg, right trunk, left trunk, right arm, left arm, and head) by converting masses to volumes assuming fat density of 0.9 g/mL and lean mass density of 1.1 g/mL. Total tissue volume included lean and fat, but not bone.

T1-weighted MRI scans without contrast were also acquired in a subset of participants at 0 and 26 weeks using the same scanner (IRM Siemens Magnetom Aera 1.5 T). Scanning covered bone anatomical landmarks at proximal and distal ends of the target area, and an oblique scan plane of 5-mm thickness with up to 100 slices was used. All scans were blinded prior to analysis. For volume calculation, IDEAL fat (Dixon sequence) images were visualized using volumetric software (Syngo.via, Siemens Healthineers, Germany). Morphology segmentation was performed through computation of watershed gradients. Tissues (fat, muscle, bone, and blood vessel) were manually defined and software was used to generate a surrogate of tissue volume using five slices, with manual adjustments where required. Impact of treatment on overgrowth was assessed by comparative measures of the same site performed at baseline and at the end of treatment period. All DXA scans and MRI readings were centralized at the coordinating center.

### Evaluation of vascular lesions

Patients with visible cutaneous vascular lesions underwent clinical photographs using the same camera in the same room with consistent illumination and color balance. Pictures were reviewed by a single expert physician (P.V.) to assess changes in vascular lesions

### Quality of life assessment

Validated quality of life (QoL) questionnaires were administered before and after treatment (Short Form 36 [SF-36] questionnaire—French version) [12]. The SF-36 instrument includes eight domains reflecting physical functioning, social functioning, vitality, role limitations (physical), role limitations (emotional), mental health, general health, and bodily pain. Each domain score ranges from 0 to 100 [13]. A higher score means a better functioning or less limitations. Scores obtained from each domain are weighted and summed to generate two summary scores, namely a physical component score (PCS) and a mental component score (MCS).

### Dosing regimen and pharmacokinetics

Taselisib dose regimen, 1 mg/day in the first 6 patients and 2 mg/day thereafter, was based on (1) an observed 50% reduction of abnormal signaling induced by a taselisib concentration of 4 nmol/L in preclinical studies in affected cells, and (2) data from a population pharmacokinetic (PopPK) model derived from over 500 cancer patients and healthy volunteers showing that the lowest expected concentration of taselisib given a taselisib dose of 2 mg every other day (eod) was 6.07 nmol/l (data from manufacturer; not published). For this reason, a mean plasma steady state concentration target around 4 nmol/L was selected as likely to be achieved with lower doses (1 mg or 2 mg) of taselisib than the doses used in cancer. Bioequivalence between 2 mg eod and 1 mg/day had been previously demonstrated by the manufacturer (data not published). Taselisib PK analyses were centrally performed in accredited clinical diagnostic laboratories. Taselisib treatment was not to be adapted based on plasma concentrations.

### Safety procedure analysis

AEs were identified by laboratory testing, clinical examination, or self-report, and collected from start of taselisib up to 30 days after the end of treatment. Severity was graded with the NCI-CTCAE (version 4.0). The Medical Dictionary for Regulatory Activities (MedDRA) System Organ Class and Preferred Term were used to summarize AEs with their incidence, severity, and relationship to taselisib. An AE was considered serious as defined by the internationally accepted standards if it was fatal or life threatening, caused persistent or significant disability or incapacity, required hospitalization or prolonged hospitalization, caused congenital anomalies, or any other important medical event [14]. All AEs and SAEs were reviewed weekly and adjudicated by a committee composed of members of the study team.

Patients were withdrawn from the study on their request, due to inability or failure to attend trial visits, due to pregnancy, or due to a severe/grade III AE occurring on treatment. Severe/grade III AEs included severe colitis leading to electrolyte derangements and not responding to oral or rectal corticosteroid treatment, severe hyperglycemia leading to hyperglycemic hyperosmolar nonketotic status or diabetic ketoacidosis and/or hospitalization, renal dysfunction (glomerular filtration rate [GFR] < 70 mL/min/1.73 m^2^), liver dysfunction (alanine transaminase [ALT] or aspartate aminotransferase [AST] ≥ 2 × ULN), pneumonitis/decline in respiratory reserve, or QTc prolongation (>500 ms for women, >490 ms for men).

### Statistical analyses

Main analysis was performed on all patients who received at least one dose of taselisib and as-treated analysis only for those who completed the study. Absolute volumes of affected and unaffected tissue at baseline and week 26 were compared. Relative change in tissue volume for the treated period was defined as “Relative % change = ([affected eot value – affected baseline value] – [unaffected eot value – unaffected baseline value])/(affected baseline value + unaffected baseline value)*100” expressed in percent (%). Paired comparisons of mean volumes and mean changes in volumes were performed using paired Student’s *t*-test in SAS version 9.4, with confirmation of equal variances. Additional statistical analyses of normally distributed data of equal variance were performed using single-sample Student’s paired *t*-tests and chi-squared analyses for discontinuous data. For all tests, *p* value < 0.05 was considered significant. Variables are presented as mean and standard deviation (SD) and interquartile range.

## RESULTS

### Population characteristics

**Fig. 2:**
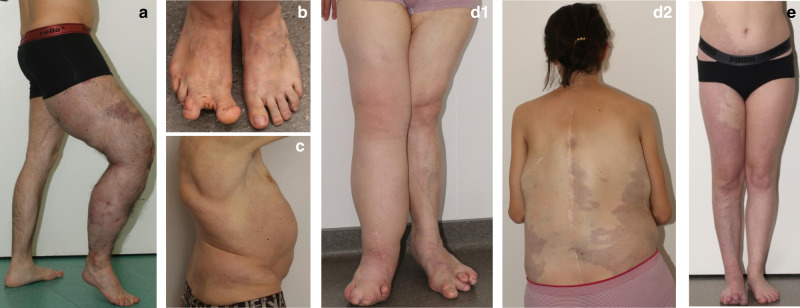
Clinical presentation of study participants with PIK3CA-related overgrowth spectrum, showing clinical heterogeneity within patients. (**a**) Patient 01-01 with a Klippel–Trenaunay syndrome (KTS) affecting predominantly the right leg. (**b**) Patient 01-02 with KTS and an amputation of the right hypertrophic second toe. (**c**) Patient 02-02 with extensive capillary malformation of trunk and extremities, with sequelae of surgery for severe scoliosis. (**d1**,**d2**) Patient 04-01 with CLOVES syndrome with extensive capillary malformation of abdomen and trunk, lymphatic deep and superficial malformation of the right inferior limb and varicose veins of left leg. (**e**) Patient 09-02 with KTS superficial capillary lymphatic malformation of right lower limb and right lower trunk.

Nineteen adults (mean age 29.4 years [SD 9.8; range 17–46]) participated between July 2017 and March 2019. Two had taken part in the PROMISE trial from June 2015 to November 2016 [6]. Two withdrew consent before receiving treatment. Among the 17 patients treated with taselisib, 6 were in the 1 mg/day cohort and 11 in the 2 mg/day cohort (see Fig. S2). Nearly half of the patients had a diagnosis of CLOVES, and the rest had KTS. Clinical characteristics of these participants are detailed in Table 1, and photographs summarizing heterogeneity of overgrowth are shown in Fig. 2 for patients that gave consent. Ten participants completed 6 months of treatment: all patients in the 1 mg/day cohort, and 4 (36%) in the 2 mg/day cohort. One patient prematurely stopped treatment after 12 weeks because of recurrent parietal abdominal abscess not related to treatment. Two patients from the 2 mg/day cohort experienced a SUSAR, leading to permanent trial interruption after review and decision by the DSMB. At time of trial interruption, the ongoing patients (*n* = 5) had been treated for at least 10 weeks.

**Table 1:** Details of genotype and phenotypic characteristics of enrolled participants.

**Patient number**	**01-01**	**01-02**	**02-01**	**06-01**	**02-02**	**04-01**	**09-01**	**16-01**	**01-03**
Sex	M	F	M	F	M	F	F	F	M
*PIK3CA* variant	c.1624G>A	c.1624G>A	c.3140A>G	c.1633G>A	c.311C>T	c.328_330delGAA	c.1133G>A	c.1035T>A	c.1258T>C
	p.(Glu542Lys)	p.(Glu542Lys)	p.(His1047Arg)	p.(Glu545Lys)	p.(Pro104Leu)	p.(Glu110del)	p.(Cys378Tyr)	p.(Asn345Lys)	p.(Cys420Arg)
General presentation	KTS	KTS	CLOVES	KTS	CLOVES	CLOVES	KTS	CLOVES	CLOVES
Fibroadipose or soft tissue overgrowth	Y	Y	Y	Y	Y	Y	Y	Y	Y
Affected areas of overgrowth	Right leg	Right foot	Right calf, right foot	Left leg	Legs	Right leg, right trunk	Thighs, arms	Trunk	Lower trunk, lower limbs
Regional lipohypoplasia (affected areas)	N	N	N	Y	Y	N	N	Y	Y
Type of vascular Malformations	Deep and superficial lymphatic malformation (right leg and pelvis)	Pelvic vascular agenesis/malformation	Varicose veins	Lymphatic malformation (left leg); capillary malformation (4th toe); varicose veins (right buttock)	Extensive capillary malformation (trunk and extremities); varicose veins	Extensive capillary malformation (abdomen and trunk), lymphatic deep and superficial malformation (right lower limb), varicose veins (left leg)	Capillary malformations (legs, arms, trunk and lower limbs), lymphedema	N	Lymphatic deep and superficial malformation (lower trunk and lower limbs)
Thromboembolic disease	Y	Y	N	Y	N	Y	N	N	Y
Hemorrhage	N	Rectal bleeding	N	Vaginal bleeding	N	N	Abundant menstruations, short cycles	N	N
Epidermal nevus	N	N	N	N	N	N	N	N	N
Extremities	Overgrowth of right foot	Overgrowth of right toe	Overgrowth of right plantar and toe; plantar connective tissue hamartoma	Overgrowth of left foot	Overgrowth of feet	Overgrowth of right toe	Bilateral and symmetrical brachymetacarpy (4th and 5th segments) and brachymetatarsy (short toes with short and buried nails)	Enlarged feet with wide spaced toes	Y
Spine	N	N	N	N	Kyphosis	Scoliosis	N	Scoliosis	Scoliosis
Others	Limited leg extension	N	N	Recurrent soft tissue infections	N	Left knee algodystrophy,	Body hypertrophy, bilateral conductive deafness, leg length discrepancy, left knee pain	N	Leukocytoclastic vasculitis, glomerulonephritis, recurrent soft tissue infections
BMI (kg/m^2^)	25.2	25.5	24.3	31.9	27.7	25.0	29.7	23.7	33.6
OFC (cm)	ND	ND	ND	55	57	55	54.5	54	ND
Development anomalies	N	N	N	N	N	Learning disabilities	N	N	N
Procedures/surgery	Venous embolization and ethanol sclerotherapy	Toe amputation	Amputation (all left foot’s toes, 2nd and 3rd metatarsals), soft tissue reduction, and liposuction	Multiple debulking surgeries	Multiple surgeries (debulking, vascular, cutaneous, spine, venous stripping)	N	N	Right toes amputation (2nd to 4th), plantar soft tissue ablation; multiple abdominal liposuctions; urgent median laparotomy for *S. aureus* sepsis	N
Overall improvement	Reduction of pain (slight), improvement: muscle and joint flexibility, better comfort when driving a car	Reduction: digestive hemorrhage (very important), pain (important), antalgic intake, correction of chronic deep anemia leading to disappearance of chronic asthenia and withdrawal of chronic blood transfusions	Reduction of: hypertrophy (-1 cm diameter on affected foot compared to baseline) pain, antalgic intake	Reduction: pain (significant), antalgic intake, asthenia, headaches, vaginal bleeding (very important)	N	Reduction: hypertrophy (−2 cm diameter on affected leg compared to baseline)	Not evaluated (early termination because of drug-related adverse event)	Slight esthetic improvement of aspect of lipoma	Reduction of left calf volume, reported better overall general state
						Pain (important), antalgic intake			
			Improvement: walking and standing durations, QoL	Improvement: walking and standing durations, QoL, social life and mood		Improvements: walking and standing durations, QoL			
		Important pain reduction with diminution of antalgic intake)							

**Patient number**	**08-01**	**11-01**	**06-02**	**12-01**	**02-03**	**04-02**	**09-02**	**16-02**
Sex	F	F	F	F	F	M	F	F
*PIK3CA* variant	c.1258T>C	c.1093G>A	c.3140A>G	c.1624G>A	c.3062A>G	c.3129G>A	c.353G>A	c.3140A>G
	p.(Cys420Arg)	p.(Glu365Lys)	p.(His1047Arg)	p.(Glu542Lys)	p.(Tyr1021Cys)	p.(Met1043Ile)	p.(Gly118Asp)	p.(His1047Arg)
General presentation	KTS	KTS	KTS	KTS	KTS	KTS with facial infiltrative lipomatosis	KTS	KTS
Fibroadipose or soft tissue overgrowth	Y	Y	N	Y	Not assessable (due to overweight)	Y	Y	N
Affected areas of overgrowth	Right leg	Left lower limb with buried nails, left face, both hands	Left leg with bony overgrowth	Left leg, left foot	Right leg, left leg	Lower limbs, hemiface	Right lower trunk, right lower limb	Left upper limb
Regional lipohypoplasia (affected areas)	N	N	N	N	N	N	N	N
Type of vascular malformations	Capillary and lymphatic malformation, with skin lymphangiectasia and purple nevus flammeus from right buttock to feet	Superficial capillary malformation, arteriovenous malformation, nevus roseus, phlebectasia	Capillary keratotic lymphangiectasia	Capillary lymphatic malformation of left leg, left foot and skin lymphagectasias	Extensive superficial capillary malformation (nevus roseus) and bilateral lymphoedema (both legs) and microcystic malformation	Extensive superficial capillary malformation (nevus roseus) and bilateral lymphoedema (both legs)	Superficial capillary lymphatic malformation of right lower limb and right lower trunk, skin lymphangiectasias of lower back	Microcystic lymphatic malformation with skin lymphangiectasias
				Cutaneous hemangiomas, and varicose veins of left leg				
Thromboembolic disease	N	N	N	Y	Y	N	N	N
Hemorrhage	N	N	N	Menorrhagia, history of rectal bleeding	Abundant menstruation	N	N	N
Epidermal nevus	N	N	N	N	Y	N	N	N
Extremities	Lymphatic malformation of 3rd, 4th, and 5th right toes	Hypertrophy of left foot, both hands	N	Lymphatic malformation, soft tissue hyperplasia with debulking surgery	Macrodactyly of right middle finger, left index finger	Superficial capillary malformation (nevus roseus) and bilateral lymphedema	Capillary malformation, with sequels of debulking surgery	Muscular retraction of muscular compartments with left hand arthrogryposis
Spine	N	N	N	N	Lordosis	N	N	N
Others	Erysipelas	N	Chronic migraine	Subcutaneous lymphangiectasis, edema of outer lips of vagina with associated lymphatic collection	Arthrosis, hypothyroidism with goiter, left jugular vein hypoplasia, anemia, spleen hamartoma, mucosal and skin lesions/infections (fungal infection, vaginal mycosis, eczema, lichen)	N	N	Left elbow flexion deformity
BMI (kg/m^2^)	21.5	24.9	18.2	21.2	60.8	32.3	30.1	19.2
OFC (cm)	57.5	57	54	ND	58	59.5	ND	53.5
Development anomalies	N	N	N	N	N	Behavioral disorder	N	N
Procedures/surgery	N	Left femoral and tibial osteotomy, cervix conization	N	Left foot (2nd toe ablation, ablation of 1st and 3rd nails)	N	Bilateral carpal tunnel	Right foot debulking surgery between 2nd and 3rd toe	N
Overall improvement*	N	Reduction of left leg volume (can wear jeans); significant pain reduction, improved physical (started jogging) and psychological condition (stopped alprazolam); disappearance of headaches	Pain reduction	Pain reduction	Reduction of leg edema and induration, muscle cramps and muscle pain, asthenia, mucosal and skin infections (no new infection with taselib); functional improvement (recovery of left knee flexion, disappearance of trigger knee), recovery of daily activity, walking range increased, mood improvement	Not evaluated because of drug-related adverse effect leading to patient’s withdrawal	Important reduction of angioma; stabilization of pre-existing varicose vein (right knee) at baseline; disappearance of pain, antalgic intake stopped walking range strongly improved improvement of mood	Disappearance of pain Improvement in quality of life; diminution of asthenia, mainly because of disappearance of night awakening due to pain

*BMI* body mass index, *KTL* Klippel–Trenaunay syndrome, *OFC* occipital frontal circumference, *QoL* quality of life.

### Safety and tolerability

All patients treated experienced at least one AE, and all but one patient had at least one AE deemed related to treatment. Overall, 48% (114/236) of AE were related to taselisib (see Table 2). The most common drug-related AEs were digestive disorders (41/114, 36%), followed by neurologic events (19/114, 17%). Eight SAEs occurred in five patients including three grade 3 drug-related AEs. More AEs related to taselisib were observed in the 1 mg cohort (51/90, 57% vs. 63/146, 43% in the 1 mg and 2 mg cohort respectively, *p* = 0.04). Patient 01-01 (included in the 1 mg cohort) alone experienced 17 grade I and 7 grade II AE considered to be related to taselisib. The primary outcome (DLT defined as drug-related toxicity ≥ grade 3 occurring in the first month of treatment) was reached at 2 mg/day of taselisib. An episode of ileitis occurred in patient 09-01 at 26 days (D26) of treatment and lasted for 25 days. This episode had been preceded by an episode of gastritis on D11, and led to permanent discontinuation. The two other SAEs were a parvovirus B19 infection on D58 (patient 16-01), which resolved within 9 days, and pachymeningitis on D26 (patient 04-02).

**Table 2:** Overview of adverse events (AEs) and serious adverse events (SAEs).

	**Intention-to-treat population (*n*** **=** **17)**
Total number of AEs recorded	236 AEs in 17/17 (100%) participants
	122/236 AEs (52%) unrelated to taselisib
At least one taselisib-related AE (all grades)	16/17 (94%)
At least one event of ≥ grade 3 severity	6/17 (35%)
Death (grade 5)	0 (0%)
At least one SAE	5/17 (29%)
At least one SAE and ≥ grade 3 severity	4/17 (24%)
At least one event leading to permanent discontinuation of taselisib	3/17 (18%)
Most frequent (>5%) taselisib-related AEs (all grades)	
Gastrointestinal	41/114 (36%)
Neurologic	19/114 (17%)
General	15/114 (13%)
Infectious	13/114 (11%)
Blood and lymphatic	10/114 (8%)

For this last AE, clinical presentation started with intense headaches, followed by seizures, cognitive impairment and gait disorder. Experimental treatment was withheld and lumbar puncture found aseptic lymphocytic meningitis. MRI showed pachymeningitis with mild meningeal hemorrhage. The participant was then withdrawn from the study. Improvement without full recovery was obtained with corticosteroid therapy (CS). Memory impairment and gait disorder persisted at 6 months on maintenance CS therapy (5 mg/day). Brain MRI performed at 3 months was suggestive of possible scars from pre-existing vascular malformations.

### Efficacy outcomes

#### Clinical improvement

**Fig. 3:**
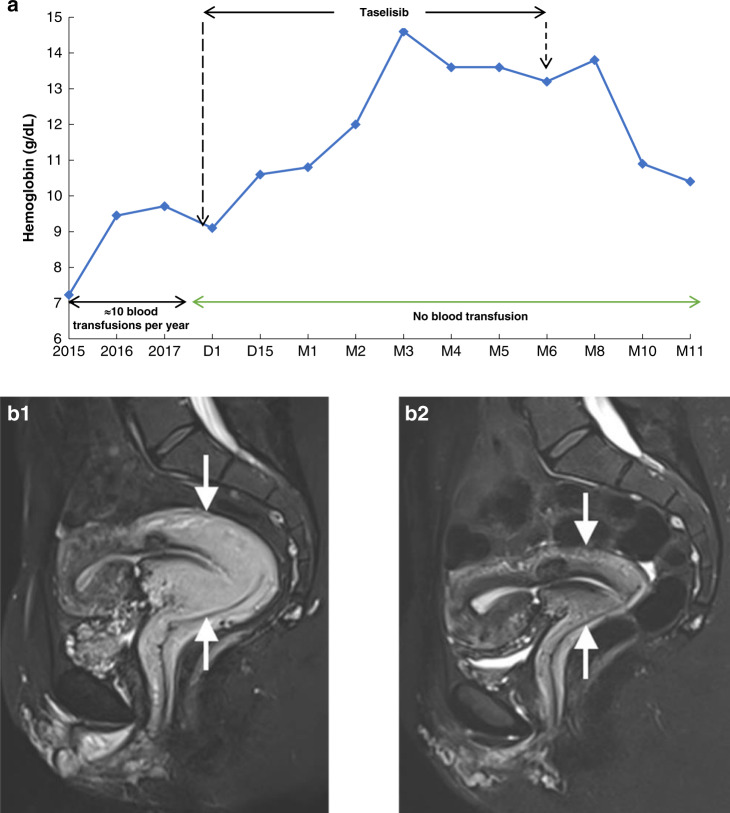
Clinical improvements in patients 01-02 and 06-02. (**a**) Evolution of hemoglobin levels and transfusion needs during the treatment period in patient 01-02. (**b1**,**b2**) Sagittal T2 TSE weighted images of the pelvis with fat saturation before and after treatment in patient 06-01. **b1**: Important circumferential hypersignal thickening (arrows) of the cervix, isthmus and proximal part of the corpus of the uterus corresponding to a uterine vascular malformation. **b2**: Images after treatment showing frank decrease in size of the womb thickening (arrows) testifying to excellent response to treatment.

All patients were clinically evaluated. The mean measured circumference of lesions remained stable after treatment compared to baseline, with a reduction of only -1.48% (SD 3.79; *p* = 0.39) (Fig. S2-A). Among the 17 patients treated, 13 (76.4%) reported at least one type of clinical improvement, while 2 reported no effect (Table 1). The main observed benefit was pain reduction or cessation in 11 patients (64.7%), leading to analgesic drug tapering or withdrawal in 4 patients. Five (29.4%) patients also had improved standing posture or walking ability, and 2 (11.7%) reported cosmetic improvement. Patient 01-02 experienced complete resolution of rectal bleeding from a pelvic vascular malformation, resulting in normalization of hemoglobin levels (Fig. 3a). She had required monthly blood transfusions for several years to maintain a 9 g/dL hemoglobin (Hb) value at baseline. After 1 month on taselisib, Hb level increased to 11 g/dL, and subsequently plateaued at a stable level of 14 g/dL Hb value from 3 months of treatment onward. Treatment interruption after 8 months (6 months of trial followed by 2 months of compassionate use), led to a rapid fall to 10 mg/dL Hb within 2 months because bleeding had resumed. Patient 06-02, who had major vaginal bleeding at baseline due to vascular hyperplasia of the uterus, was similarly improved with taselisib, with cessation of vaginal bleeding, associated with reduction of uterus volume (Fig. 3b1 and b2).

### Imaging

Soft tissue from affected and, whenever possible, unaffected body regions were measured at 0 and 26 weeks by DXA and MRI. Among 17 treated patients, 13 (76.4%) were evaluated on DXA, and 11 (64.7%) on MRI. Patients who experienced 2 SUSARs could not undergo DXA or MRI scans at the end of treatment and were excluded from efficacy analysis. DXA was not feasible in one patient with morbid obesity, and anatomy of two patients did not allow comparison of affected versus unaffected tissue on DXA. Volumetric MRI could not be performed at local hospitals for two patients after trial interruption.

At baseline, the median total tissue volume at affected sites was 2,705 ml (interquartile range [IQR]: 210–11,820). The mean decrease in total tissue volume at affected sites was −4.2% (*p* = 0.81; SD 14.01, range −8.9 to +4.3) assessed by volumetric MRI (Fig. S2-B). No significant changes in affected tissue (+0.94%, SD 7.87; *p* = 0.50) were observed on DXA. A trend toward increase of fat of 5.9% (SD 13.62, *p* = 0.09) was observed in affected tissue but not in unaffected tissue (3.8%, SD 19.19, *p* = 0.27) (Table S2 and S3). Per protocol, analysis restricted to nine analyzable patients who completed the study found similar results, with a nonsignificant increase of 3.4% of mean total volume in affected tissue (SD 6.65, *p* = 0.14).

Patients treated with 2 mg/day of taselisib exhibited a slight decrease in affected tissue (−1.3%, SD 2.74), although not statistically significant (*p* = 0.21).

### Quality of life

QoL analysis was performed in 16/17 (94.1%) patients. Patient 04-02, who presented with cognitive impairment at time of evaluation, could not be analyzed. QoL scores before and after taselisib treatment did not significantly differ (Table S4). MCS was not significantly modified by the intervention (44.3 after treatment vs. 46.2 before treatment, *p* = 0.44). Evaluation of the physical component showed significant improvement of the dimension “limitations due to physical status” (the higher the score, the better the performance) (67.2 [SD 41.6] after treatment vs. 42.2 [SD 33.8] at baseline, *p* = 0.037), although not reflected statistically on the overall PCS (*p* = 0.091).

### Blood testing

No clinically significant changes in blood parameters were observed, except an increase in Hb (12.19 ± 0.38 g/dL before treatment vs. 12.91 ± 0.38 after treatment, *p* = 0.029) (see Table S5).

## DISCUSSION

Authors of the present study are not aware of any other published clinical trial evaluating the safety and efficacy of a targeted inhibitor of the PI3K alpha subunit in patients with PROS. Our results show that, despite clinical improvements such as pain reduction, cessation of chronic bleeding or improved QoL, the negative safety profile makes long-term use of taselisib inappropriate in PROS.

Taselisib use led to two SUSARs, triggering early termination of our trial. This is at odds with the recent report of Venot et al., who claimed a favorable safety profile of another PI3K-alpha subunit inhibitor, alpelisib, in a cohort of 19 PROS patients [7]. This discrepancy may be attributable in part to the more systematic reporting of AE in our study, in accordance with good clinical practice, documenting severity, type of event, incidence, and relationship with taselisib [15]. The profile of vascular/overgrowth phenotypes, and disease severity, also differed between the two studies. Last but not least, differences may be explained by a difference in selectivity for the p110α catalytic subunit of PI3K encodes by *PIK3CA*. Alpelisib is reported to have higher selectivity than taselisib; taselisib also inhibits the PI3Kγ and δ catalytic subunits of PI3K, which likely modulate the immune system and possibly explains the inflammatory adverse events that we report [16].

This trial was interrupted due to occurrence of two SUSARs. For enteritis, there is little doubt about the causal relationship with taselisib therapy, since the event occurred after a rechallenging with taselisib; however, it is debatable that pachymeningitis with meningeal hemorrhage was related to the drug treatment. A complication of a pre-existing vascular malformation cannot be ruled out, since cerebral imaging was not performed prior to treatment in this patient. Spontaneous disease progression is also a possibility. Those observations strongly advocate for extensive assessment of the phenotype at baseline, to avoid missing any asymptomatic vascular anomalies in PROS patients.

One of the challenges of therapeutic trials in PROS patients with a CLOVES/KTS phenotype is the proper assessment of safety and efficacy profile of candidate drugs in the long term. Surprisingly, none of the patients included in our study experienced hyperglycemia, whereas it has been described in 50–65% of patients on PI3K inhibitors included in cancer trials [17–19]. In the study by Venot et al. [7], 3 of 19 patients had transient and mild hyperglycemia. If this observation supports a dose–effect relationship, our follow-up period was still relatively short. Because those treatments may have to be used on a long-term basis, delayed side effects, as well as resistance to treatment, may occur long after treatment initiation [20].

Another challenge in PROS trials is the choice of criteria used to assess efficacy. While the gold standard in clinical trials may usually be summarized as “an objective endpoint for each outcome,” this may not be suitable in PROS patients due to their clinically heterogeneous presentation. Biological markers as potential endpoints or surrogate markers are an interesting possibility to investigate, but the profile of any such marker across the natural history of PIK3CA-related disease would have to be established first. We performed a post hoc analysis separately in patients with vascular and lipomatous phenotypes who completed the study. Taselisib had no effect on blood levels of D-dimers and adiponectin respectively, but the study was not designed to answer this specific question. Studies assessing these biological markers’ behavior in PIK3CA-related diseases are needed to validate possible endpoints for future therapeutic trials. Here, nonsignificant results for objective quantification of overgrowth on imaging are in marked contrast with the clinical improvement reported by patients. Patient-reported outcomes (PROs) may be a more relevant metric of efficacy in diseases with heterogeneous or complex phenotype, particularly in rare diseases, where reaching statistical significance is a challenge due to small cohorts and heterogeneity of phenotypes. Moreover, the clinical improvements observed here using PRO contrast with the nonsignificance of standardized evaluation with the SF-36 questionnaire. Again, this discrepancy raises the difficulty of QoL investigation through quantitative questionnaires in in rare disease trials, where statistical significance is difficult to obtain. Nevertheless, it would be hazardous to consider PRO as self-sufficient to evaluate efficacy in PROS patients. Several patients reported functional improvement, particularly pain reduction, but without visible anatomical effect. This advocates for pursuing a composite evaluation in PROS, using both objective and subjective outcomes.

As knowledge of the genetic and molecular mechanisms of PIK3CA-related overgrowth improves, candidate drugs are increasingly being considered for this indication. Given the relatively low number of affected patients, sequential treatment with different drugs in the same patient is not uncommon, be it through clinical trials or compassionate use. From our experience, it seems that subsets of clinical presentations (such as exclusive fatty tissue overgrowth, or lymphatic or capillary vascular malformations) may be associated with better efficacy of a particular therapeutic class. Several patients who were sequentially treated with sirolimus then taselisib in our PROMISE and TOTEM trials showed a heterogeneous clinical response. Despite several attempts to date to evaluate drugs inhibiting the PI3K/AKT/mTOR signaling pathway, none of them have been shown to have a positive risk to benefit ratio in PROS patients. A phase I trial in patients with Proteus syndrome with pan-AKT-inhibitor miransertib is ongoing [21]. This experimental drug could be assessed in CLOVES patients as well [22]. It is likely that in the future, rather than a “one-size-fits-all” treatment approach, panels or a combination of various drugs will be used [23]. Hopefully, as the number of patients with PROS included in therapeutic trials increases, a tailored approach facilitated by identification of clinical patterns associated with better drug-specific response may have a major impact on PROS therapy.

## Conclusion

The registered and approved TOTEM trial showed that low-dose taselisib has an unfavorable safety profile in KTS and CLOVES, despite promising individually observed clinical effects. Our study emphasizes the need for close monitoring of safety in patients treated with this class of medications under compassionate use. Further clinical trials should be conducted for other inhibitors of the PI3K/AKT/mTOR signaling pathway in PROS patients.

## Supplementary information

Supplementary Information

## Data Availability

Data can be supplied upon individual request to the corresponding author.
