# Supplementary material for: Safety and efficacy of low-dose PI3K inhibitor taselisib in adult patients with CLOVES and Klippel–Trenaunay syndrome (KTS): the TOTEM trial, a phase 1/2 multicenter, open-label, single-arm study
Source: Genet Med. 2021 Aug 12;23(12):2433–42. doi: 10.1038/s41436-021-01290-y (PMC8631579; doi:10.1038/s41436-021-01290-y)

Supplemental material

Table S1. Inclusion and exclusion criteria (full version)

| **Inclusion Criteria**   - Have given, or their legal representative has given, written informed consent to participate - Aged 16 years to 65 years inclusive - Male or female - Post-zygotic PIK3CA variant - Clinically stable in the opinion of the investigator - Participant Pregnancy and contraception: - Female participants of child bearing potential must use an effective method of contraception during treatment and for at least 3 months after the final dose of Taselisib.   - Acceptable methods are:     - True abstinence (this must be the participant’s usual and preferred lifestyle, not just for the duration of the trial)     - Oral contraceptive (either combined or progestogen alone)     - Contraceptive implant, injections or patches     - Vaginal ring     - Intrauterine device (IUD, coil or intrauterine system)     - Condom and cap     - Diaphragm plus spermicide   - A female participant of child bearing potential is defined as a sexually mature woman not surgically sterilized or not post-menopausal for at least 12 consecutive months if aged 55 years or older. - Men must use one of the following, reliable forms to contraception for the entire duration of treatment and for 3 months after the final dose of taselisib:   - Condom plus spermicide even if female partner is using another method of contraception (Men should also use a condom to protect male partners, or female partners who are pregnant or breast feeding, from exposure to the Trial medicine in semen).   - True abstinence (this must be the participant’s usual and preferred lifestyle, not just for the duration of the Trial) |
| --- |
| **Exclusion Criteria**   - Pregnant or breastfeeding - HIV infection - Hypersensitivity to Taselisib or any of its excipients - Any current medical disorder or medication likely to impair ability to follow the trial protocol safely and Effectively - Are concurrently taking an mTOR inhibitor or any other small molecule inhibitor of the PI3K-AKT signaling pathway - Unable or unwilling to give informed consent - Sirolimus or Taselisib treatment in 12 weeks prior to screening - Treatment with a strong inducer or inhibitor of CYP3A4 without the possibility to stop this medication within the week prior to the screening. This includes:   - Macrolide Antibiotics: clarithromycin, telithromycin, erythromycin, troleandomycin   - Gastrointestinal prokinetic agents: metoclopramide.   - Antifungals: itraconazole, ketoconazole, fluconazole, voriconazole, clotrimazole   - Calcium channel blockers: verapamil, diltiazem, nicardipine   - Grapefruit containing foods/drinks   - Anticonvulsants: carbamazepine, phenobarbital, phenytoin   - Antibiotics: rifampicin, rifabutin, rifapentine   - Herbal preparations: St. John’s wort (Hypericum perforatum). Other drugs: bromocriptine, cimetidine, danazol, cyclosporine, lansoprazole, calcium containing antacids. - Inability to attend trial visits - If less than 3 months post- major surgery at screening - Any past medical history of inflammatory bowel disease or chronic diarrhoea of unknown aetiology - History of type 1 or type 2 diabetes mellitus requiring insulin, GLP-1 analogues or oral hypoglycaemic agents. - History of inflammatory bowel disease, ischemic colitis, or colitis of unknown origin. - Fasting blood glucose > 6.9 mmol/l - HbA1C > 6% - Long QT, congenital or acquired - Active pneumonitis - Patients who require daily supplemental oxygen - Inadequate renal function defined as creatinine clearance or radioisotope GFR < 70ml/min/1.73 m2 - Inadequate liver function defined as:   - Total bilirubin > 2.0 x ULN or conjugated bilirubin > 2.0 xULN for age, and   - SGPT (ALT) or SGOT (AST) ≥ 1.5 x ULN for age, and   - Serum albumin < 30 g/L - Inadequate fasting LDL cholesterol > 4.2 mmol/l - Deprived of freedom by an administrative or court order, or benefiting from a system of legal protection (tutorship, curatorship or safeguard of justice). - Not covered by health insurance |

**Table S2. DXA-measured differences in tissue volume in affected and unaffected tissues. Total tissue = lean + fatty tissue.**

| **Tissue Type** | **Mean percentage change in tissue volume (SD) N=13** | | | **P value for mean percent change** |
| --- | --- | --- | --- | --- |
|  | **Affected** | **Unaffected** | **Δ** |  |
| **Total** | 0.94 (7.87) | 1.52 (9.09) | -0.58 (7.71) | 0.79 |
|  |  |  |  |  |
| **Fat** | 5.86 (13.62) | 3.78 (19.19) | 2.07 (20.53) | 0.72 |
|  |  |  |  |  |
| **Lean** | -1.54 (6.62) | -0.47 (6.84) | -1.07 (6.77) | 0.58 |
|  |  |  |  |  |

**Table S3 : DXA (n=13) and MRI (n=11) individual assessments**

|  | DXA | | | | | | | | | | | | | | | | | | | | |  | | MRI | | | | | | | |
| --- | --- | --- | --- | --- | --- | --- | --- | --- | --- | --- | --- | --- | --- | --- | --- | --- | --- | --- | --- | --- | --- | --- | --- | --- | --- | --- | --- | --- | --- | --- | --- |
|  |  |  | Total mass (kg) | | | | | Fat mass (kg) | | | | | | Lean mass (kg) | | | | | | | |  | | Total volume (mm^3^) | | | | | | | |
|  |  |  | Affected | | Unaffected | |  | Affected | | Unaffected | |  | Affected | | | Unaffected | | |  | |  | |  | |  | |  | |  | |  |
| Subject ID | affected site | Unaffected site (control) | baseline | eot | baseline | eot | relative  % change | baseline | eot | baseline | eot | relative  % change | | baseline | eot | | baseline | eot | | relative % change | |  | | Assessed site | | Baseline | | Eot | | relative  % change | |
| 01-01 | right lower limb | Left lower limb | 17.2 | 19.6 | 9.9 | 10.8 | 6.3 | 5.3 | 7.1 | 2.1 | 2.3 | 21.0 | | 11.6 | 12.2 | | 7.3 | 8.0 | | -0.5 | |  | | right lower limb | | 5180.0 | | 4260.0 | | -17.8 | |
| 01-02 | 2 lower limbs | 2 upper limbs | 27.8 | 29.9 | 7.8 | 9.0 | 2.9 | 13.7 | 15.6 | 3.3 | 4.4 | 5.0 | | 13.3 | 13.5 | | 4.2 | 4.3 | | 0.5 | |  | | right foot | | 210.0 | | 201.0 | | -4.3 | |
| 02-01 | right lower limb | Left lower limb | 13.4 | 13.6 | 12.1 | 12.1 | 1.0 | 4.1 | 4.1 | 2.2 | 2.3 | -1.0 | | 8.8 | 9.0 | | 9.3 | 9.2 | | 1.9 | |  | | Right leg, right foot | | 1695.0 | | 1594.0 | | -6.0 | |
| 04-01 | 2 lower limbs | 2 upper limbs | 41.9 | 42.1 | 3.5 | 3.9 | 0.2 | 11.4 | 13.7 | 0.7 | 1.0 | 16.2 | | 29.6 | 27.6 | | 2.5 | 2.7 | | -6.9 | |  | | right lower limb, left leg | | 11820.0 | | 10305.0 | | -12.8 | |
| 06-01 | left lower limb | right lower limb | 13.5 | 13.5 | 12.8 | 12.4 | -0.8 | 5.9 | 5.7 | 5.3 | 5.1 | -4.4 | | 7.2 | 7.4 | | 7.0 | 6.8 | | 2.5 | |  | | left leg and foot | | 2705.0 | | 2600.0 | | -3.9 | |
| 02-02 | all parts affected | no unaffected region | - | - | - | - | - | - | - | - | - | - | | - | - | | - | - | | - | |  | | left foot | | 163.0 | | 209.0 | | 28.2 | |
| 16-01 | 2 lower limbs | 2 upper limbs | 25.0 | 21.5 | 4.7 | 4.5 | -11.4 | 6.2 | 5.8 | 1.2 | 1.3 | -7.0 | | 17.9 | 14.9 | | 3.2 | 3.0 | | -13.3 | |  | | - | | - | | - | | - | |
| 09-01 | not evaluated - early withdrawal | - | - | - | - | - | - | - | - | - | - | - | | - | - | | - | - | | - | |  | | not evaluated - early withdrawal | | - | | - | | - | |
| 01-03 | trunk | 2 upper limbs | 59.3 | 61.8 | 6.3 | 5.7 | 5.4 | 10.9 | 12.9 | 0.9 | 0.6 | 20.2 | | 47.2 | 47.7 | | 5.1 | 4.8 | | 1.5 | |  | | abdominal fat | | 16728.0 | | 15283.0 | | -8.6 | |
| 16-02 | left arm | right arm | 4.4 | 3.4 | 2.6 | 2.5 | 0.5 | 1.2 | 1.3 | 0.9 | 0.8 | 6.0 | | 2.1 | 2.0 | | 1.6 | 1.6 | | -3.1 | |  | | - | | - | | - | | - | |
| 08-01 | right lower limb | Left lower limb | 11.1 | 12.1 | 10.4 | 11.3 | 0.4 | 3.8 | 4.0 | 3.8 | 4.1 | -0.4 | | 7.1 | 7.7 | | 6.3 | 6.8 | | 0.9 | |  | | right leg | | 1235.0 | | 1362.0 | | 10.3 | |

**Table S3 (end): individual DXA (n=13) and MRI (n=11) individual assessments**

|  | DXA | | | | | | | | | | | | | | | | | |  | MRI | | | |
| --- | --- | --- | --- | --- | --- | --- | --- | --- | --- | --- | --- | --- | --- | --- | --- | --- | --- | --- | --- | --- | --- | --- | --- |
|  |  |  | Total mass (kg) | | | | | Fat mass (kg) | | | | | | Lean mass (kg) | | | | |  | Total volume (mm^3^) | | | |
|  |  |  | Affected | | Unaffected | |  | Affected | | Unaffected | |  | | Affected | | Unaffected | |  |  |  |  |  |  |
| Subject ID | affected site | control | baseline | eot | baseline | eot | relative  % change | baseline | eot | baseline | eot | | relative % change | baseline | eot | baseline | eot | relative  % change |  | affected site | Baseline | Eot | relative  % change |
| 06-02 | right inferior limb | Left inferior limb | 8.3 | 8.8 | 8.0 | 8.4 | 0.9 | 3.0 | 3.4 | 2.6 | 2.9 | | 1.8 | 5.0 | 5.1 | 5.1 | 5.1 | 0.3 |  | - | - | - | - |
| 09-02 | right inferior limb | Left inferior limb | 8.0 | 7.8 | 9.2 | 8.5 | 2.9 | 3.1 | 3.1 | 3.6 | 3.3 | | 3.3 | 4.6 | 4.4 | 5.3 | 4.8 | 2.5 |  | overgrowth at posterior face of right knee | 11.5 | 8.7 | -24.5 |
| 02-03 | not evaluated - morbid obesity | - | - | - | - | - | - | - | - | - | - | | - | - | - | - | - | - |  | left leg | 12938.0 | 12182.0 | -5.8 |
| 11-01 | left leg | right leg | 16.8 | 16.1 | 12.6 | 12.7 | -3.1 | 7.3 | 7.1 | 4.9 | 5.1 | | -2.5 | 9.1 | 8.5 | 7.2 | 7.2 | -3.6 |  | left lower limb | 3648.0 | 3604.0 | -1.2 |
| 12-01 | left leg | right leg | 11.2 | 10.3 | 10.3 | 9.2 | 0.6 | 3.8 | 3.2 | 3.0 | 2.9 | | -6.0 | 7.1 | 6.7 | 6.9 | 5.9 | 4.3 |  | - | - | - | - |
| 04-02 | not evaluated - early withdrawal | - | - | - | - | - | - | - | - | - | - | | - | - | - | - | - | - |  | not evaluated - early withdrawal | - | - | - |

DXA: dual energy X-ray absorptiometry; MRI: magnetic resonance imaging; eot : end of treatment; Rules for DXA analysis: unaffected site must be completely unaffected; If one limb is affected, compare with contralateral unaffected; If both limbs are equally affected, compare with both unaffected limbs; If both limbs affected with one predominant side, compare the limb that is most affected and compare with the unaffected limb on the same side; If thorax is affected on both sides, compare with unaffected sites where possible e.g. both legs, both arms; If diffusely affected, state no unaffected sites.

Relative % change is estimated by the formula: ((affected eot value - affected baseline value) – (unaffected eot value - unaffected baseline value) / (affected baseline value + unaffected baseline value))*100

**Table S4- Quality of life assessment using the SF-36 short form (n=16)**

|  | *Baseline* | *End of treatment* | *delta* | *Range* | *p-value* |
| --- | --- | --- | --- | --- | --- |
| **Mental Component Summary,** mean (SD) | 46.62 (11.22) | 44.26 (11.68) | -2.36 | (-28.55 - 14.12) | 0.44 |
| **Physical Component Summary,** mean (SD) | 35.51 (9.26) | 40.96 (10.23) | 5.45 | (-26.75 - 19.18) | 0.09 |
| *Physical Function* | 58.13 (23.66) | 61.56 (28.33) | 3.44 | (-100 - 40) | 0.66 |
| *Role Physical* | 42.19 (33.81) | 67.19 (41.55) | 25.00 | (-100 - 100) | **0.04** |
| *Bodily Pain* | 35.88 (23.32) | 50.88 (26.36) | 15.00 | (-72 - 84) | 0.14 |
| *General Health* | 42 (22.35) | 41.88 (17.48) | -0.13 | (-55 - 37) | 0.98 |
| *Vitality* | 43.02 (17.12) | 49.06 (20.10) | 6.04 | (-23.33 - 40) | 0.23 |
| *Social Function* | 61.72 (28.31) | 59.38 (22.13) | -2.34 | (-100 - 37.5) | 0.78 |
| *Role emotional* | 75 (37.52) | 72.92 (36.96) | -2.08 | (-100 - 66.67) | 0.88 |
| *Mental Health* | 61.84 (21.78) | 59.13 (22.77) | -2.72 | (-64 - 22.5) | 0.64 |
| *SD: Standard deviation* |  |  |  |  |  |

**Table S5. Comparison of biologic parameters after 6 months of treatment vs. before treatment by taselisib (n=15)***

| Biological data | | Baseline (n=15) | End of treatment (n=15) | Normal range | p-value** |
| --- | --- | --- | --- | --- | --- |
| ***Hematology***, mean ± SD | |  |  |  |  |
| red blood cells (10^6^/mm^3^) | | 4.19 ±0.44 | 4.47 ±0.56 | 4.5 - 6.5 | **0.006^a^** |
| Haemoglobin (g/dL) | | 12.2 ± 0.4 | 12.9 ±0.4 | 13.0 - 17.0 | **0.029^a^** |
| Haematocrit (%) | | 37.1 (0.9) | 39.1 (0.9) | 40.0 - 54.0 | **0.014^a^** |
| Mean Corpuscular Volume (µ^3^) | | 88.8 (5.6) | 87.9 (4.9) | 80.0 - 100.0 | 0.349 |
| Mean Corpuscular Hemoglobin (pg) | | 29.1 (0.7) | 29.0 (0.5) | 27.0 - 32.0 | 0.757 |
| Red cell Distribution Width (%) | | 14.5 (3.5) | 14.1 (1.8) | 0 - 15 | 0.460 |
| Platelets (10^3^/mm^3^) | | 225 (81) | 235 (53) | 150 – 450 | 0.663 |
| Mean Platelets Volume (µ^3^) | | 10.5 (0.30) | 10.50 (0.27) | **-** | **0.039^b^** |
| Leucocytes (10^3^/mm^3^) | | 5.86 (1.59) | 5.37 (1.31) | 4.0 10-0 | 0.552 |
| Neutrophils (10^3^/mm^3^) | | 3.18 (0.31) | 2.81 (0.22) | 1.8 – 7.5 | 0.326 |
| Lymphocyts (10^3^/mm^3^) | | 1.77 (0.17) | 1.80 (0.12) | 1.0 – 4.0 | 0.805 |
| Monocytes (10^3^/mm^3^) | | 0.50 (0.04) | 0.45 (0.03) | 0.2 – 1.0 | 0.139 |
| Eosinophils (10^3^/mm^3^) | | 0.26 (0.25) | 0.26 (0.20) | 0 – 0.8 | 0.313 |
| Basophils (10^3^/mm^3^) | | 0.04 (0.00) | 0.04 (0.00) | 0 – 0.2 | 0.564 |
| aPTT ratio | | 3.41 (9.17) | 1.92 (3.09) | <1.20 | 0.115 |
| Prothrombin Time (%) | | 86 (2) | 90 (2) | >70 | 0.064 |
|  | |  |  |  |  |
| ***Biochemistry***, mean ± SD | |  |  |  |  |
| Sodium (mmol/L) | | 140 (2) | 139 (1) | 133 – 143 | 0.067 |
| Potassium (mmol/L) | | 3.7 (0.3) | 3.9 (0.4) | 3.4 – 4.6 | 0.603 |
| Urea (mmol/L) | | 0.31 (0.07) | 0.29 (0.07) | 2.5 – 6.4 | 0.346 |
| Creatinin (µmol/L) | | 6.75 (1.43) | 7.44 (2.45) | 59 - 104 | 0.012 |
| GFR (mL/min/1.73m^2^) ^#^ | | 121.92 (4.99) | 111.69 (6.25) | > 90 | **0.041^b^** |
| total protein (g/L) | | 73 (1) | 74 (1) | 67 - 85 | 0.285 |
| Total CL (mmol/L) | | 1.51 (0.29) | 1.55 (0.33) | 3.10 – 5.70 | 0.604 |
| LDL (mmol/L) | | 0.83 (0.25) | 0.83 (0.28) | - | 0.989 |
| HDL (mmol/L) | | 0.50 (0.13) | 0.52 (0.15) | 1.04 – 1.55 | 0.476 |
| TG (mmol/L) | | 0.91 (0.61) | 0.98 (0.70) | 0.50 – 1.70 | 0.245 |
| Glucose (mmol/L) | | 0.78 (0.10) | 0.81 (0.06) | 4.30 – 5.90 | 0.115 |
| HbA1C (%) | | 5.5 (0.3) | 5.5 (0.3) | 4.0 – 6.0 | 0.840 |
| C-peptide (ng/mL) | | 1.8 (0.8) | 1.9 (0.5) | 0.5 – 3.0 | 0.266 |
| Adiponectin (µg/mL) | | 8.26 (2.09) | 11.7 (3.84) | **-** | **0.010^b^** |
| Leptin (ng/mL) | | 10.23 (5.50) | 13.00 (13.1) | - | 0.645 |
| Fibrinogen (g/L) | | 3.5 (1.4) | 3.3 (0.9) | 2.0 -4.0 | 0.607 |
| D-Dimer (ng/mL) | | 2681 (5016) | 1872 (2379) | <500 | 0.460 |
|  | |  |  |  |  |
| ***Hepatic fonction***, mean ± SD | |  |  |  |  |
| Total bilirubin (µmol/L) | | 7 (4) | 6 (2) | 3 - 14 | 0.876 |
| SGOT (UI/L) | | 14 (5) | 18 (6) | 15 - 37 | **0.000^b^** |
| SGPT (UI/L) | | 22 (16) | 24 (15) | 16 - 61 | 0.321 |
| Alkaline Phosphatase (UI/L) | | 77 (26) | 68 (17) | 45.0 – 117.0 | 0.214 |
|  | aPTT : Actived Partial Thromboplastin Time ; CL : cholesterol; LDL : low density lipoprotein; HDL: high density lipoprotein; TG : triglycerides; HbA1C : glycated haemoglobin; SGOT: Serum Glutamic Oxaloacetic Transaminase ; SGPT: Serum Glutamate Pyruvate Transaminase  * 2 patients excluded from analysis (early termination for SUSAR) **student test for paired data or wilcoxon rank test  ^#^ renal clearance calculated with CKD-EPI formula  a. clinically significant variation from the investigator’s point of view; b. not clinically significant variation from the investigator’s point of view | | | | |

**Figure S1. Overview of study design and 3+3 dose escalation rules**
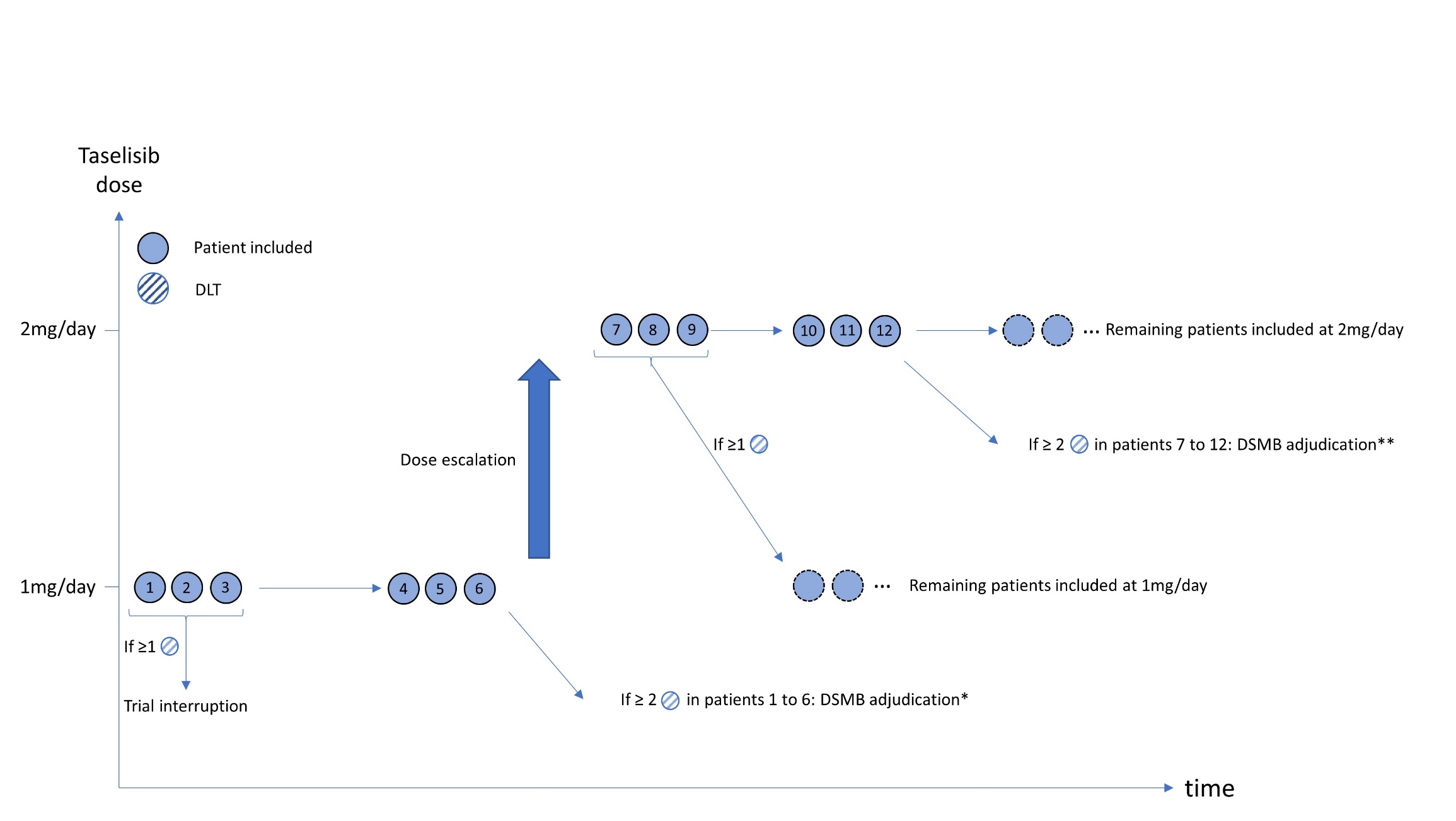


DLT: Dose Limiting toxicity; DSMB: Data Safety Monitoring Board

**Figure S2. Effect of Treatment on Overgrowth Measurements**. A: estimated overall perimeter change (cm)~~(24)~~ clinically measured at affected sites during study period. Values at baseline are obtained by summing all measures of all affected sites at baseline. The same method was applied for end of treatment values. B: MRI-estimated overall volume change (cm^3^) for each patient at affected sites during study period.

A.


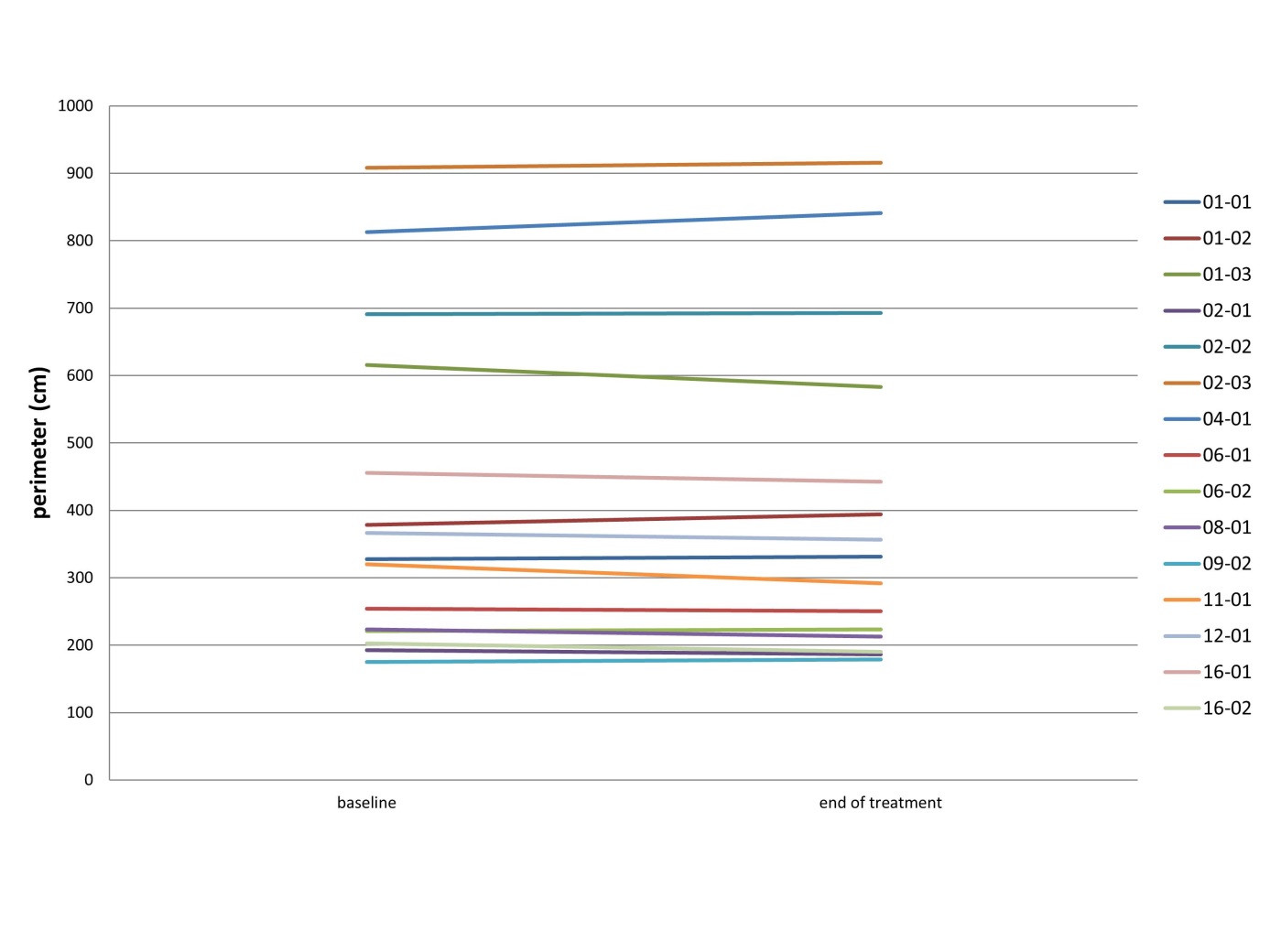


B.


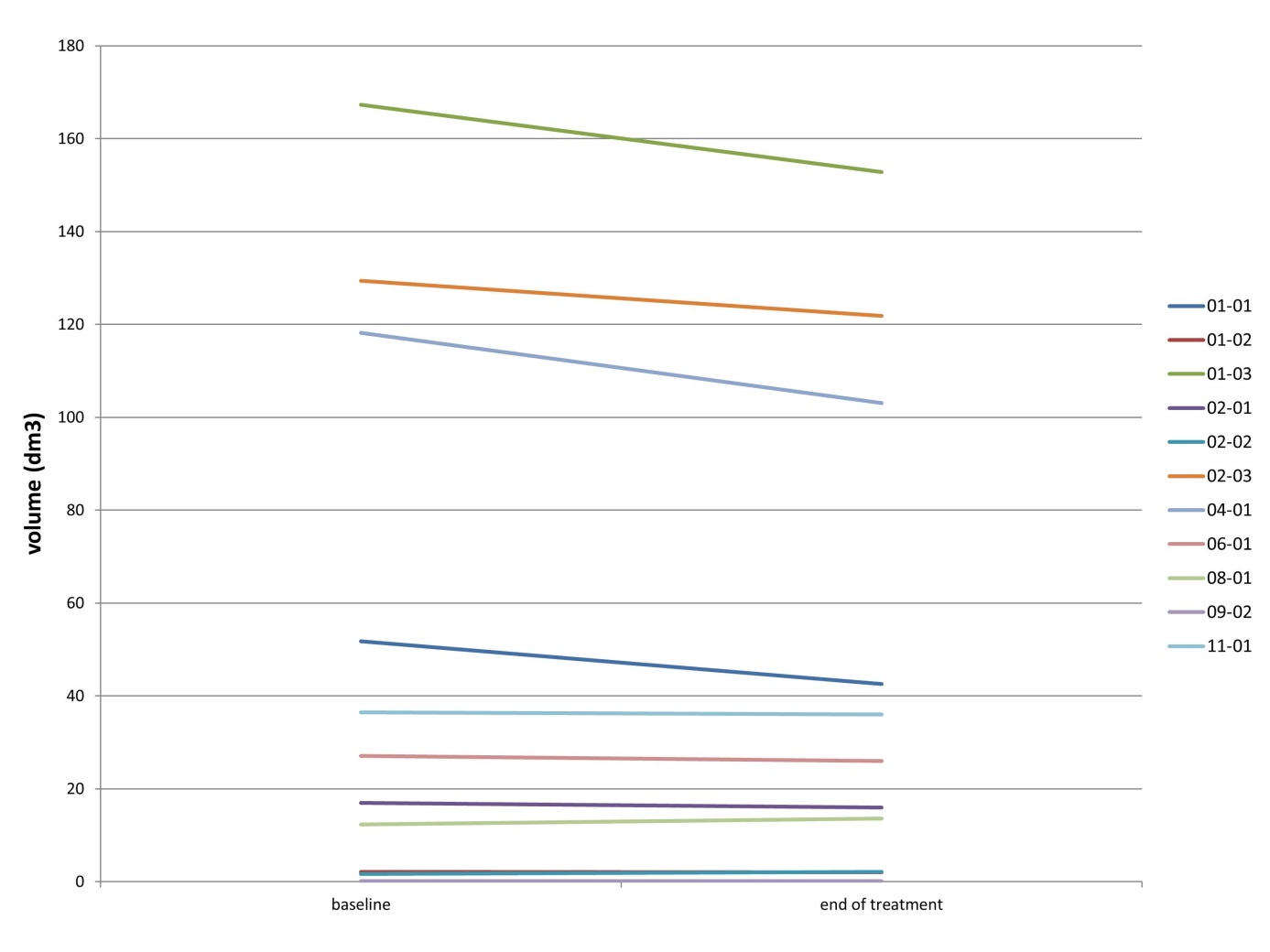

Supplement: Supplementary file 1 — Supplementary Information [file 41436_2021_1290_MOESM1_ESM.docx]
